# Targeting tumour microenvironment by tyrosine kinase inhibitor

**DOI:** 10.1186/s12943-018-0800-6

**Published:** 2018-02-19

**Authors:** Hor-Yue Tan, Ning Wang, Wing Lam, Wei Guo, Yibin Feng, Yung-Chi Cheng

**Affiliations:** 1 School of Chinese Medicine, Li Ka Shing Faculty of Medicine, The University of Hong Kong, Hong Kong, People’s Republic of China; 2 Department of Pharmacology, Yale University School of Medicine, New Haven, CT USA

**Keywords:** Tumour microenvironment, Tyrosine kinase inhibitors, Immunotherapy

## Abstract

Tumour microenvironment (TME) is a key determinant of tumour growth and metastasis. TME could be very different for each type and location of tumour and TME may change constantly during tumour growth. Multiple counterparts in surrounding microenvironment including mesenchymal-, hematopoietic-originated cells as well as non-cellular components affect TME. Thus, therapeutics that can disrupt the tumour-favouring microenvironment should be further explored for cancer therapy. Previous efforts in unravelling the dysregulated mechanisms of TME components has identified numerous protein tyrosine kinases, while its corresponding inhibitors have demonstrated potent modulatory effect on TME. Recent works have demonstrated that beyond the direct action on cancer cells, tyrosine kinase inhibitors (TKIs) have been implicated in inactivation or normalization of dysregulated TME components leading to cancer regression. Either through re-sensitizing the tumour cells or reversing the immunological tolerance microenvironment, the emergence of these TME modulatory mechanism of TKIs supports the combinatory use of TKIs with current chemotherapy or immunotherapy for cancer therapy. Therefore, an appropriate understanding on TME modulation by TKIs may offer another mode of action of TKIs for cancer treatment. This review highlights mode of kinase activation or paracrine ligand production from TME components and summarises the findings on the potential use of various TKIs on regulating TME components. At last, the combination use of current TKIs with immunotherapy in the perspectives of efficacy and safety are discussed.

## Background

Protein phosphorylation, one of the most prevalent post-translational modification of protein, is tightly regulated by specific protein kinase that transfer phosphate group to the amino acid residue [1]. To our knowledge, there are in total 518 kinases in human genome [2], while 90 of them are classified in the category of tyrosine kinases (58 of them are receptor tyrosine kinase RTK; the remaining are non-receptor tyrosine kinase) [3]. Specifically, tyrosine kinase transfers phosphate group from ATP to tyrosine residue of the protein. In carcinogenesis, the aberrant activation of protein phosphorylation, particularly by RTKs has been frequently described [4]. Mutation or gene amplification of tyrosine kinase signalling further promote the carcinogenesis process, including survival, proliferation, motility, and metabolism as well as escape from immune surveillance [5]. For instances, overexpression of epidermal growth factor receptor (EGFR) and platelet derived growth factor receptor α/β (PDGFR) have been well implicated in supporting various malignancy growth and progression [6, 7].

The binding of ligand such as growth factors or cytokines to the extracellular domains of RTKs initiate the signalling cascade by changing its structure and kinase activation. Whereas the non-RTKs which lack of extracellular domains mainly serves as the downstream effector of RTKs [8]. Understanding on the mode of action of tyrosine kinases had led to discovery of many small molecule inhibitors proved to be effective for the cancer treatment. To date, more than 30 RTK inhibitors have been approved by US FDA. They can inhibit single target or multiple targets. For instances, gefitinib and erlotinib which primarily inhibit EGFR, is used for EGFR-mutated lung cancer patients [9]. Imatinib mesylate has multiple targets, c-KIT, PDGFR and c-ABL, is indicated for acute and chronic myeloid leukemic as well as gastrointestinal stromal patients [10, 11]; sorafenib which multi-targeted VEGFR, PDGFR and Raf, is utilized for advanced renal cell carcinoma and hepatocellular carcinoma patients [12, 13]. Much previous efforts on tyrosine kinase inhibitors (TKIs) have focused on their direct actions in regulating tumour growth and angiogenesis, while recent emerging studies have re-focused on how TKIs modulate the tumour microenvironment (TME). An appropriate understanding on how TKIs alter the TME may help pave another mode of action of TKIs in cancer therapy. To maximally exploit the therapeutic benefit of current tyrosine kinase inhibitors, we systematically searched through the PubMed database with the MeSH terms of “tumour microenvironment” and “Protein-Tyrosine Kinases/antagonists and inhibitors”. In this review, we highlight the impact of kinase activation and paracrine production of ligand from tumour stroma, and summarise the findings on the potential effect of various type of tyrosine kinase inhibitors on tumour microenvironment components which including mesenchymal cells, hematopoietic cells and non-cellular components. The perspective of combination use of current kinase inhibitor with immunotherapy and modulation of TME in overcoming TKIs resistance for cancer treatment are also discussed.

## Targeting tumour microenvironment with TKI

Kinase inhibition in tumour cells by tyrosine kinase inhibitors offers promising clinical benefit to cancer patients, especially, who have tumour with mutated kinases [14]. Nonetheless, aberrant activation of tumour microenvironment may fail therapeutics that are merely targeting on cancer cells. The fact that tumour cells can promote their growth by recruiting and communicating with other type of cells, such as mesenchymal- and hematopoietic-originated cells, in the tumour microenvironment (TME) [15]. Given the accumulating evidences of tyrosine kinase paracrine receptor activation or ligand production by TME components, TKIs may be a promising TME modulator apart from regulating the intrinsic functions of tumour cell [16–18]. Taken the example of tumour immunology, TKIs generally act on the two mechanisms: immunogenic control and immune conditioning [19]. Immunogenic control is the modulation of tumour cells sensitivity in response to alteration of immunosuppressive phenotype, whereas immune conditioning is the direct changes of TKIs incurred on the function and number of immune cells populations. As detailed below, TKIs have been implicated in essentially all components of TME and thus increasingly recognised as a promising candidate for TME modulation.

## Targeting the mesenchymal cells

### Endothelial cells

Endothelial cells are known to play pivotal role in tumour neovascularization. The role of TKIs in endothelial cells has been identified either through direct action on endothelial cells or through mediating the communication between endothelial cells and tumour cells. Earlier studies observed the PKI 166, epidermal growth factor receptor (EGFR) inhibitor attenuated EGFR activation in tumour associated endothelial cells and caused cell apoptosis. The action is deemed to be EGFR-specific as it was not observed on EGFR-negative mice [20]. Subsequent findings on erlotinib and gefitinib, EGFR inhibitors showed combination use of TKI with chemotherapeutic agents enhanced vessel permeability and fractional plasma volume, which consequently, optimized the efficacy of drug delivery [21, 22]. Endothelial cells secreted-EGF promoted the migration property of T-cadherin deficient cancer cells, which was found to be more sensitive to EGF response. Gefitinib treatment blocked the cancer cell migration, suggestive of the negative correlation of cancer cells-expressing T-cadherin and EGFR activation on endothelial cells [23]. More specifically, gefitinib reduced the coverage of endothelial cells and perivascular cells as well the recruitment of perivascular progenitor cells into tumour region [24]. Moreover, endothelial cells are known to express receptor tyrosine kinases vascular endothelial growth factors (VEGFR) and platelet-derived growth factors (PDGFR), in which binding of VEGF or PDGF to VEGFR and PDGFR on endothelial cells elicits endothelial cells proliferation, migration and tumour neovascularization [25]. Thus, there has been considerable efforts in targeting VEGFR and PDGFR on endothelial cells as ideal therapeutic target for tumour neovascularization. For instances, SU5416, the VEGFR2 inhibitor eliminated melanoma microvasculature [26]; imatinib mesylate, the PDGFR inhibitor blocked the angiogenesis in prostate cancer bone metastases [27]; Ki8751, the VEGFR inhibitor reversed MDR1 up-regulation and endothelial cells chemo-resistance [28].

Given that several target receptors act as endothelial cell regulators, multi-targeted tyrosine kinase inhibitors are also being evaluated for better therapeutic outcome. The dual tyrosine kinase inhibitor AEE788 is utilized to inhibit EGFR and VEGFR binding on endothelial cells and resulted in apoptosis in both tumour and endothelial cells [29]. The intervention of pazopanib that targeted on PDGFR, VEGFR and KIT receptors in endothelial cells enhanced chemo-sensitivity of cancer cells to chemotherapeutic treatment [30]. Sunitinib, the inhibitor for VEGFR and PDGFR inhibited tube formation and outgrowth of aortic ring [31] as well as induced endothelial cells death [32]. Observing the shared constitutively activated tyrosine kinases of PDGFR and Src, the use of dasatinib, the Src/Abl kinase inhibitor also deferred tumour angiogenesis [33]. Taken together, aiming at PDGFR and VEGFR on endothelial cells and pericytes appear to be an ideal target for vessel normalization; however, imatinib and sunitinib treatments have also shown to increase the dissemination of tumour cells. Recent finding demonstrated that vasculature-supporting pericytes exhaustion enhanced breast-to-lung metastasis, which is associated with shift of hypoxia and epithelial-to-mesenchymal phenotype. Therefore, suppressing pericyte coverage with imatinib and sunitinib further increased the metastatic rate followed by enhanced hypoxia and mesenchymal phenotype in tumour-bearing mice [34]. By targeting on endothelial cells and pericytes which preserve the vessel integrity, the combination use of TKIs with other interventions could be a better approach to achieve the optimal tumour inhibitory function. For instance, the MET inhibitor PF2341066 was administrated with imatinib or sunitinib to reduce the epithelial-to-mesenchymal transition [15] and co-administration of imatinib with osteoclast-depleting zoledronate declined prevalence of lymph node metastasis [35].

### Fibroblast

Cancer associated stromal cell, comprised of approximately 40% of total tumour volume [36], plays a pivotal role in carcinogenesis, in which its role involves promoting cancer cell initiation [37], extracellular matrix remodelling [38], and even preserving stem/progenitor cells [39]. For instances, mesenchymal stromal cells protect the stem/progenitor cells from TKI-induced apoptosis, thereby leading to reduced sensitivity of stem cells towards TKI exposure and tumour relapse [39]. The negative implication of TKI on cancer associated fibroblast was further demonstrated through co-culturing of tumour cells with α-SMA-stained tumour stromal cells, in which significant increase of stromal cell while decline of tumour cell growth following gefitinib treatment. This phenomenon postulates the intrinsic resistance of cancer-associated stromal cells to gefitinib [40]. Recent study analysing the tumour epithelium and stroma in cholangiocarcinoma observed the association of the tumour stroma mediated gene signature and poor patient prognosis. Interestingly, tumour epithelium was associated with HER2 deregulation and aberrant expressions of EGFR and HGFR followed by upregulation of inflammatory cytokines in stroma regions. Treatment with lapatinib, the inhibitor targeted on HER2 and EGFR reprogrammed the tumour microenvironment which favoured of cholangiocarcinoma cell growth suppression [36]. The spleen tyrosine kinase (SYK) has been identified as the B-cell receptor and inhibition of SYK critically attenuated B cells maturation and survival [41]. Besides targeting on BCR signalling, SYK inhibitor R406 restricted secretion of adhesion factors CXCL12 and VCAM-1 as well as Mcl-1, the chemo resistant factor by stromal cells, which in turn resulted in resistant chronic lymphocytic leukemic cell death [42]. By silencing the receptor tyrosine kinases expressed on fibroblast cells such as FAK and VEGFR, PF562271 [43] and regorafenib treatment [44] reduced populations of fibroblast and interrupting the interaction of tumour cells with stromal cells, resulted in cancer regression. Tumour cells was shown to produce a pro-inflammatory factor, leukaemia inhibitor factor (LIF) upon TGFβ exposure and further promoted JAK-mediated actomyosin contractility in cancer-associated fibroblast and reprogramming of extracellular matrix towards pro-invasive phenotype. Accordingly, JAK2 tyrosine kinase inhibitor, ruxolitinib eradicated the remodeling towards pro-invasive phenotype by fibroblast, suggested the JAK inhibition as ideal target for fibroblast inactivation [45]. The phenomenon has also been documented in cancer cells resistance towards TKIs, where addition of roxulitinib re-sensitized the cancer cells towards TKIs exposure [46].

Fibroblast/stromal cells highly expressed PDGF receptors; production of PDGF ligands by cancerous cells triggered the up-regulation of fibroblast growth factors (FGF) by fibroblast which favours of tumour angiogenesis and proliferation. Notably, bone marrow stromal cells highly express PDGFRβ, while minimally detected in cancer cells. Therefore, there are emerging strategies targeting PDGFR or FGF on fibroblast in regressing tumour cell growth. For instances, inhibition of PDGFR by imatinib declined the secretion of FGF-2 and FGF-7 by cancer associated fibroblast resulted in reduced tumour progression [47]; combination treatment of nilotinib, PDGFR inhibitor and everolimus, MTOR inhibitor reduced fibroblast and cancer growth [48]; sunitinib treatment abrogated the bone marrow stromal cells growth and adhesion components that facilitate their interactions to tumour cells, thereby interrupting the tumour cells homing and colonization to bone [49]; dovitinib, the multikinase inhibitor attenuated the cross-talk between stromal and epithelial cells mediated by FGFR and regressed prostate cancer growth and bone metastasis [50]. A similar observation was found in primary colonic fibroblast, in which sunitinib attenuated its growth via PDGFβ signalling [51]. On the other hand, FGF2 from tumour microenvironment also have shown to confer resistance of cancer cells towards imatinib; addition of ponatinib, the multikinase inhibitor of BCR-ABL and FGFR re-sensitized the cancer cells [52]. Similar observation of reversing the cancer cell resistance was obtained by addition of PD-173074, FGFR inhibitor to lapanitib treatment [53].

The acquired resistance of tumour cells towards TKI has been frequently reported [46, 54]. The mechanism involved in drug resistance can be diverse, ranging from cell extrinsic to intrinsic conditions. One of the well-known mechanisms inducing resistance is activation/phosphorylation of Met, the proto-oncogene on cancer cells [55]. Apart from acquiring the resistance upon constitutive Met activation in cancer cells, current findings suggested that ligand dependent paracrine receptor activation from TME components initiates the tyrosine kinase inhibitor resistance. Co-culturing of hepatocyte growth factor (HGF), the Met ligand-secreting fibroblast and cancer cells declined cancer cell sensitivity towards TKI treatment [56]. HGF has been shown to highly express in EGFR-TKI resistant lung cancer patients [57]. While co-treatment with another c-MET TKIs, crizotinib [58, 59] or NK4 [60] reversed the drug resistance. Very interestingly, endothelial cells produced EGFR ligand also initiated the resistance of lung cancer cells towards crizotinib and co-treatment with erlotinib re-sensitized the cells towards crizotinib treatment [61]. By abolishing c-MET activation by fibroblast using DCC2701, a c-MET/TIE-2/VEGFR inhibitor, the cancer growth and migration was subsided. Besides, the HGF produced by normal ovarian fibroblast are much higher than the cancer-associated fibroblast, suggested normal neighbouring fibroblast in supporting cancer cell progression [62]. Another recent finding confirmed the paradoxical activation of cancer associated fibroblast conferred melanoma resistance towards BRAF inhibition, as observed from (a) reduced ERK/MAP kinase activity in reduced stromal density region, (b) co-culture of melanoma cells with fibroblast declined drug-induced apoptosis on melanoma cells, (c) BRAF inhibitor augmented matrix remodelling ability of fibroblast. Incorporation of FAK, Src or PDGFR inhibitors re-sensitized melanoma cells towards BRAF inhibitor in the co-culture system of tumour and stromal cells via re-activation of ERK. Collectively, the findings suggest aiming at the receptor ligand of cancer-associated stromal cells may be ideal to overcome the drug resistance. Disruption of either axis on cancer associated fibroblast with tyrosine kinase inhibitors modulate the cross-talk between tumour and stromal cells, and further augmented the anti-tumour effect [63].

### Stem cells

**Fig. 1:**
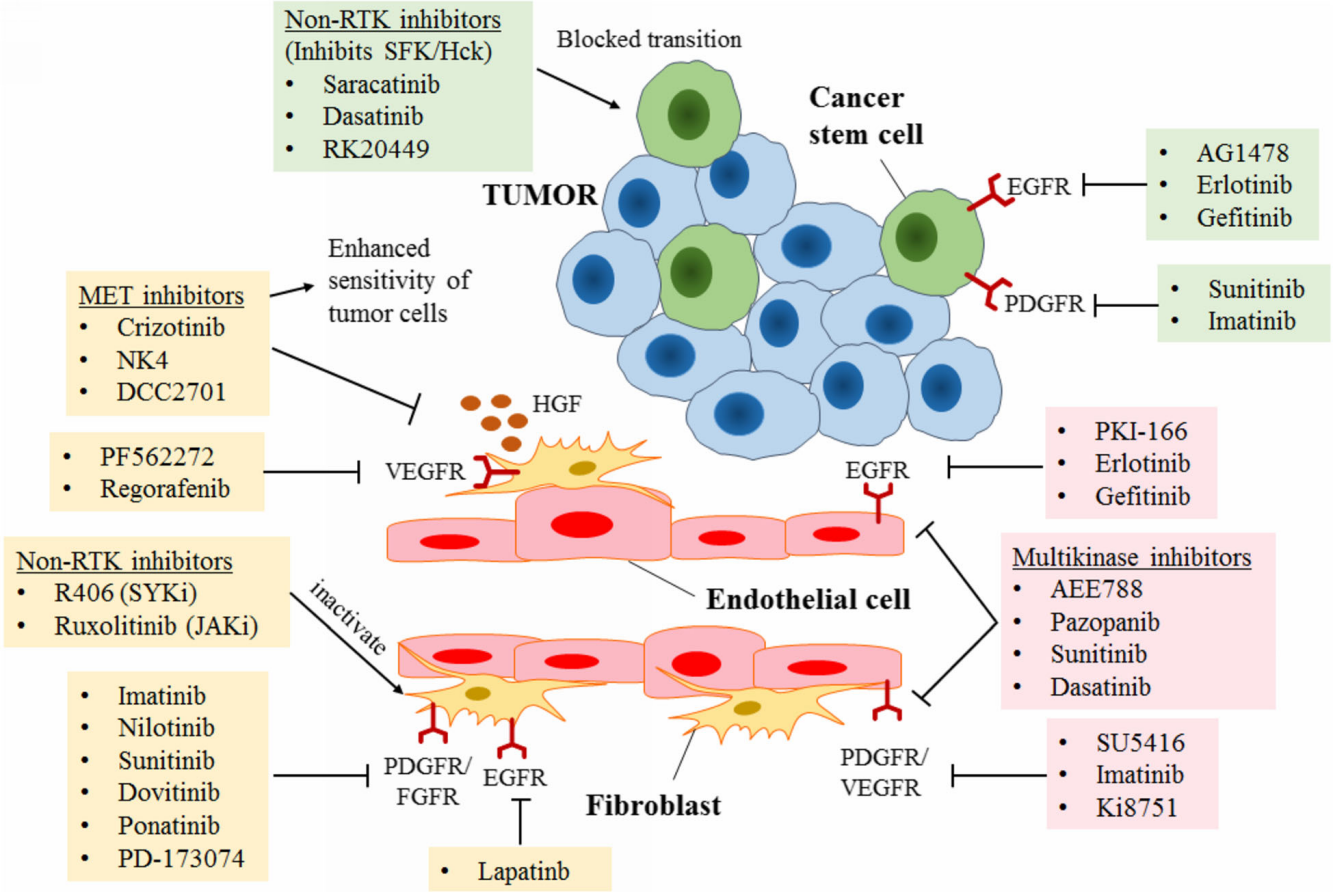
The implication of TKIs on mesenchymal-originated cells

Apart from endothelial cells and fibroblast, mesenchymal stem cells are also able to secrete EGF and express EGF receptor. Suppressing EGF receptor using AG1478 therefore suppressed mesenchymal stem cells mediated mammosphere formation [64]. Yoshida et al. proposed the phenomenon of continuous EGFR activation in EpCAM highly expressed cancer stem like cells while gefitinib treatment declined the population viability [65]. The same also reported by Lacerda et al. in which erlotinib treatment decreased expression of EGFR in mesenchymal stem cells and declined tumour metastasis [66]. In addition to EGFR, cancer stem cells have also been shown to be responsive to other TKI treatment which further triggered cancer cell death. For instances, treatment of sunitinib declined ALDH+ cancer stem like cell population and re-sensitized the cells to radiation treatment [67]; addition of dasatinib declined the mesothelial-like cell proliferation and thereby attenuated fibrillar formation in metastatic cancer cells [68]; imatinib abrogated cytokines expression that associated with bone marrow homing and leukemic hematopoietic stem cells survival [69]. Recent finding proposed the reprogramming of non-stem like cancer cells to high resistant cancer stem cells following taxane intervention and the transitions was associated with the activation of Src family kinase/Hck signalling. Notably, inhibition of SFK/Hck using RK20449 or dasatinib declined the cancer stem-like populations and allowed temporal chemotherapeutic action [70]. This phenomenon suggested targeting at SFK/Hck signalling with TKI enhanced the tolerance of cancer cells towards chemotherapeutic agents. The phenomenon also has been documented in co-culture of breast cancer cells with bone mesenchymal stem cells, where treatment of saracatinib, the SFK/Hck inhibitor on mesenchymal stem cells blunted the cancer cell migration [71]. Figure 1 summarized the TKIs that targeted on mesenchymal originated cells for subsequent cancer cell regression.

## Targeting the haematopoietic cells

### Precursor cells

Early findings postulated that the osteoblast, bone marrow precursor cells in bone marrow microenvironment tend to produce substances for instance HGF which further promotes cancer cell proliferation and migratory activities. Intervention of NK4, HGF inhibitor or imatinib mesylate declined the cancer cells functions in the presence of osteoblast [72, 73]. As a Src/Abl kinase inhibitor, dasatinib was known to inhibit the SFK kinase activity and the downstream molecule MMP9 [74]. Apart from restricting the migration of cancer cells, dasatinib also declined the infiltration of MMP9+ myeloid cells and limited their motility through down-regulation of MMP9 [75]. Inhibition of STAT3 using JAK inhibitor AZD1480 also found to decline tumour-associated myeloid population and inhibit myeloid infiltration to tumour site [76].

### Immunosuppressive cells

**Fig. 2:**
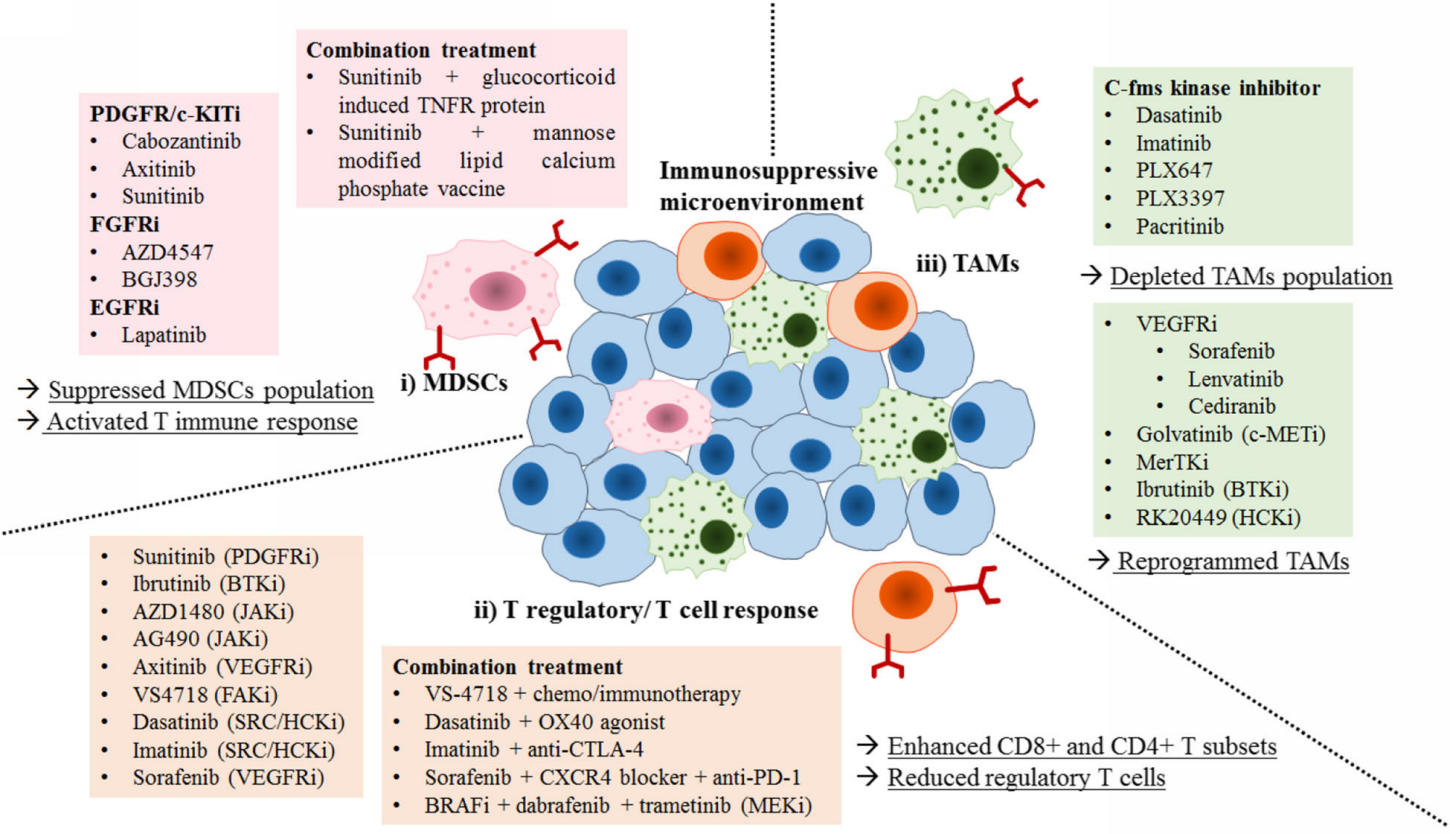
The immuno-regulatory effect of TKIs on suppressive populations in tumour microenvironment

The immunosuppressive environment, which primarily composed of myeloid-derived suppressor cells (MDSCs), regulatory T lymphocytes (Treg) and tumour associated macrophages, promoted escape of tumour cells from immune surveillance [77]. Accumulating therapeutic strategies aimed at immune-suppressive cells in tumour microenvironment to reduce immune tolerance. Evidences have emerged that TKIs switched the immunosuppressive environment and promoted the anti-tumour immunity (Fig. 2).

#### Myeloid-derived suppressor cells, MDSCs

Myeloid-derived suppressor cells MDSCs, the bone marrow derived heterogenous suppressor cells, has been shown to positively correlate to cancer progression. In advanced stage of cancer patients, tumour may induce expansion of MDSCs and in turn resulted in suppressive action of innate and adaptive immune response by high populations of MDSCs in tumour microenvironment [78]. Sunitinib treatment has found to decline MDSCs populations and MDSCs mediated regulatory T cell development in tumour tissues. Further mechanism study showed sunitinib modulated the co-stimulatory molecule and secretive factors profiles as well as composition of tumour infiltrating leukocytes favouring of T cell activation. Observing sunitinib co-operatively targeting on VEGFR2, c-kit receptor, PDGFR as well as Flt3, further investigation postulated that c-KIT is responsible on the suppressive action of sunitinib on MDSC expansion and survival [79]. Another study by Xin et al. also demonstrated the suppression of MDSCs by sunitinib via STAT3 inhibition [80], suggested the specific blockade of c-KIT or VEGFR1–3 using TKIs on MDSCs may be effective in producing a permissive immune-competent tumour microenvironment. Thus, it is not surprised observing accumulating studies on TKIs targeted at VEGFR and c-KIT in reversing the suppressive microenvironment. For instance, use of axitinib, the VEGFR inhibitor declined MDSCs while induced CD8 T cell infiltration to melanoma [81, 82]; cabozantinib, a multikinase inhibitor perturbated the immune cells subset and reduced MDSCs and regulatory T cells in spleen [83]. Moreover, the capability of sunitinib in disrupting MDSCs/Treg subsets in tumour microenvironment may enhance the response of tumour cells towards immune-stimulating therapies such as vaccines or antibodies intervention. For instances, the synergistic use of sunitinib with glucocorticoid induced TNFR related protein (GITR) increased infiltration of CD8(+) T and natural killer populations followed by repolarization of macrophages towards immuno-competent M1 phenotype that favours of tumour regression [84]. Similar observation also obtained on combination treatment of encapsulated sunitinib with mannose-modified lipid calcium phosphate vaccine on melanoma, in which the immune response was shifted towards Th1 phenotype resulted in tumour apoptosis [85]. Despite the potent effect of VEGFR/c-KIT targeted TKIs on MDSCs as reported, the resistance of MDSCs towards sunitinib treatment has also been reported and the intra-tumoral survival of MDSCs was associated with STAT5 activation and GM-CSF upregulation [86, 87]. It is supported by another observation that prolonged use of sunitinib in orthotopic glioma bearing mice triggered restoration of macrophages and Gr1 (+) MDSCs thereby promoted aggressive phenotype of tumour cells [88]. These findings suggested that single use of sunitinib may not be sufficient in attenuating development of cancer resistance and secondary therapy is needed to support the sustained immunosuppression induced by VEGFR/c-KIT targeted TKIs.

In addition to VEGFR/c-KIT as therapeutic target of MDSCs, the use of AZD4547, the FGFR inhibitor has also been proposed as a target of MDSCs action. AZD4547 reduced breast cancer proliferation and migration through declining the MDSCs population while enhancing T populations [89]. Consistent with the finding, BGJ398, another FGFR inhibitor was found to inhibit MDSCs population and tumour vasculature. Although tumour relapse was observed after 4 months, the combination uses of EGFR inhibitor, lapatinib with BGJ398 significantly deferred the tumour reversion [90]. Another prominent recent finding demonstrated that enhancement of MDSCs population in BRAF inhibitor-resistant tumour microenvironment which initially reduced after BRAF inhibitor treatment. The phenomenon was not observed in regulatory T cells, which postulated that the tumour resistance was contributed by restored MDSCs recruitment via CCL2/CCR2 regulation. Further study, nevertheless, showed solely MDSCs depletion could not re-sensitize tumour cells towards BRAF inhibitor treatment, while combination treatment with immunotherapies anti-CTLA-4 and anti PD-1 elicited tumour cells to immunotherapy-triggered cancer cell death [91].

#### T lymphocytes

Accumulating studies have related the cross-talk between MDSCs and regulatory T subset, generally, MDSCs recruited and supported the activation of T regulatory populations [92]. Thus, it is not surprising to observe MDSCs reduction by TKIs further led to regulatory T subset suppression. Nonetheless, as a matter of fact, immune microenvironment is dynamic and the responses are affected by extensive cross-talk between immune populations at different levels. The direct target of TKIs on T populations has also been widely described. Using adoptive transfer method, Kujawki et al. showed that sunitinib treatment with adoptively transferred T populations declined the shift of FoxP3 (−) T to regulatory T phenotype as well as increased CD8 (+) cytotoxic T infiltration. The phenomenon is explained as sunitinib-mediated STAT3 inactivation in T populations further enhanced the STAT3-deficient T cells infiltration [93]. Ibrutinib, an inhibitor of Bruton’s tyrosine kinase which is originally designed for inhibition of B cell malignancy [94], was found to reduce CD4 (+) T population and PD-1 expression on chronic lymphocytic leukemic patients. Further study observed dislodge of macrophages with leukemic cells as well as reduced chemo-attractant CXCL13 following ibrutinib treatment [95]. Concomitant use of ibrutinib with toll like receptor ligand also enhanced the antigen presenting property of cancer cells for T cell recognition [96]. Moreover, the JAK inhibitor, AZD1480 and AG490 has been described to reduce suppressive T populations while augment anti-tumour CD8 (+) T, M1 macrophages and natural killer populations [97, 98]. Likewise, the VEGFR inhibitor, axitinib also has been shown to increase CD4 (+) and CD8 (+) T population, while progressive patients showed increase regulatory T subset and PD-1 expression following axinitib treatment [99]. This further suggests acquiring resistance of immune cells to axitinib treatment upon disease progression. One mechanism involves the focal adhesion kinase (FAK) has been shown to regulate the transcriptional activation of chemokine and cytokines that further facilitates T cells response. The authors showed FAK-mediated paracrine secretion of CCL5 promoted regulatory T population while reduced CD8 (+) T subsets. Inhibition of FAK kinase using VS-4718 markedly augmented CD4 (+) and CD8 (+) populations while declined regulatory T subsets [100]. Another report implicated VS-4718 treatment augmented the sensitivity of tumour cells towards chemo- and immuno-therapies, primarily through declining the immunosuppressive M2 macrophages, GR1 (+) granulocytes and regulatory T populations [101].

Dasatinib, a multikinase inhibitor, reduced T cellular proliferation, its associated cytokine production and cellular response [102, 103]. The inhibitory effect of dasatinib on T cells is mediated by LCK inactivation, the member of Src family that regulates T-cell receptor TCR signalling. Consistent with the findings, recent study specifically showed dasatinib treatment reduced regulatory T populations while enhanced CD8 (+) anti-tumour T response. Co-stimulation of T response using OX40 agonist induced synergistic tumour inhibitory effect compared with dasatinib or anti-OX40 alone [104]. Consistently, imatinib mesylate, another inhibitor of Src family kinases enhanced CD8 (+) T population and cellular proliferation while declined regulatory T subset. Co-administration of imatinib with anti-CTLA-4, the T cell modulator showed greater tumour regression compared with single treatment [105]. Similar mechanism as dasatinib and imatinib has also been documented by sorafenib treatment, in which sorafenib also impaired T cells activation through LCK inactivation on T cells [106, 107]. In this regard, sorafenib treatment increased CD4 (+), CD8 (+) T population while reduced PD1 (+) CD8 (+) T as well as declined the regulatory T cellular function and proliferation [108, 109]. In line with these observations, clinical investigation on 45 sorafenib-receiving HCC patients showed reduced Th2 and regulatory T populations after high dose treatment of sorafenib (400 mg and 800 mg/day) with mean plasma concentration of 3-6 mg/L, suggested the immune-modulatory effect of sorafenib [110]. However, another contradictory finding in advanced hepatocellular carcinoma HCC postulated the increase of immunosuppressive T regulatory, M2 macrophages as well as intra-tumoral PD-L1 expression following sorafenib treatment, which is mediated by enhanced hypoxia in tumour microenvironment. Combination treatment of sorafenib with CXCR4 blocker and anti-PD-1 reprogrammed the immunosuppressive condition to anti-tumour phenotype and further induced HCC regression [111]. In support of this notion, Cabrera et al. postulated the dose-dependency of sorafenib on immuno-regulation, in which reduced IL2 secretion by CD4 (+) T cells while increased regulatory T suppressive activity above 3 μM of sorafenib treatment [112]. These findings further suggested the existence of dose response of TKIs on immune-modulation and the optimal dose sought may offer the modest clinical benefit for patients.

On the other hand, many small molecules inhibitors may acquire resistance upon disease progression whereby single treatment may not sufficient in altering the immunosuppressive tumour microenvironment. The VEGFR inhibitor, axitinib has been shown to increase CD4 (+) and CD8 (+) T population, while progressive patients showed increase regulatory T subset and PD-1 expression following axinitib treatment [99]. Likewise, BRAF inhibition initially enhanced CD8 (+) T infiltration and reduced immunosuppressive factors in melanoma patients, however, the CD8 (+) T population gradually decreased along with disease progression [113]. Combination treatment of BRAF inhibitor and MEK inhibitor, dabrafenib and trametinib re-established the T cells population, suggesting combination treatment may be beneficial for the rebound immune profile in progressed cancer. This is in accordance with the notion that MEK activation exerts cancer cell resistance while inclusion of MEK inhibitor may overcome it [114].

#### Tumour associated macrophages, TAMs

Much of the past and current efforts in eliminating the tumour associated macrophages (TAMs) have focussed on blocking TAMs survival or recruitment, inhibiting TAMs function and re-polarizing the TAMs phenotype that favour tumour regression. Typically, the c-fms kinase, or known as CSF1 receptor is a ligand-activated kinase that modulate monocytes and macrophage survival and proliferation [115]. The potent ‘off-target’ inhibitors of FMS receptor, dasatinib and imatinib have been shown to inhibit the growth of recruitment and growth of macrophages [116, 117]. Notably, imatinib treatment modestly depleted macrophage populations after 4 weeks of treatment in gastrointestinal stromal tumour. It was followed by cytokine profile shifting towards anti-tumour phenotype via activation of CCAAT/enhancer binding protein β. The TAMs phenotype re-shifted to tumour-promoting-like upon cancer cells acquired resistance towards imatinib [118]. PLX647, a FLT3 inhibitor also showed 35-fold above selectivity against its enzymatic homologs FMS at 1 μM [119]. Consistently, PLX647 treatment significantly suppressed macrophage activation and proliferation as governed by FMS. The same also observed on its counterpart compound PLX3397, in which it enhanced the sensitivity of cancer cells towards chemotherapeutic treatment through declining TAMs infiltration to tumor microenvironment [120]. Observing the homologue of CSF1R and FLT3, pacritinib, the JAK2/FLT3 inhibitor, has also been shown to interfere CSF-1R signaling. Treatment of pacritinib on nurse-like cell, the monocyte-like cells in chronic lymphocytic leukemia CLL led to nurse-like cell death and CLL regression [121]. Although CSF1R-targeted TKIs may potently deplete macrophages, it is arguable that systemic blockade of all macrophage populations may be detrimental.

Therefore, apart from macrophage depletion, therapeutic strategies in re-programming TAMs immunosuppressive phenotype towards anti-tumour profile by TKIs intervention have also emerged as potent TAMs-targeted cancer therapy. For instances, sorafenib treatment stimulated IL12, IL18 and IL6 production in TAMs after LPS priming, which further supported natural killer cells function [122]. Tie2 receptor has been shown to highly express on monocytes/macrophages and its paracrine secretion supports tumour neovascularization [123]. Study by Nakazawa et al. postulated that the c-MET inhibitor golvatinib, which also interfere the Ang-Tie2 and EphB4-EphrinB2 signalling, reprogrammed TAMs phenotype towards M1 anti-tumour-like. The golvatinib treatment also reduced macrophage infiltration into tumour sites via Ang-Tie2 blockade [124]. Besides, the authors also demonstrated combination treatment of golvatinib with lenvatinib, the VEGF inhibitor sensitized cancer cells towards lenvatinib treatment, further justified the previous study that Ang2 inhibition reversed the cancer resistance of VEGF inhibitor [125]. Likewise, the combination treatment of cediranib, pan-VEGFR inhibitor with anti-Ang-2 altered TAMs phenotypes, further led to vessel normalization in glioblastoma [126]. As one of the members in the family of TAMs associated receptor tyrosine kinases (RTK), MerTK has been previously shown to mediate the primary functions of macrophages -to engulf and phagocytize bacteria [127]. In this regard, macrophages in human glioblastoma highly expressed MerTK while UNC20205, the MerTK inhibitor treatment declined M2 macrophage population in glioma associated microenvironment [128]. Likewise, MerTK inhibitor also decreased M2 macrophage associated TGFβ secretion, thereby led to reduced tumour metastasis [129].

Accumulating studies postulate the BTK was activated/phosphorylated upon Toll like receptors (TLR) stimulation [130], and activation of BTK was associated with TLR3-associated cytokine production in macrophages [131]. Treatment of ibrutinib declined chemokines CXCL12, CXCL13, CCL19 and VEGF production by TAMs, which in turn resulted in declined invasion and migration of lymphoid cells [132]. Likewise, ibrutinib also enhanced the anti-tumour phenotype of TAMs in chronic lymphocytic leukemia [133]. Recent study also postulated that the hematopoietic cell kinase (HCK), which belongs to the Src kinase family is constitutively expressed on myeloid cells and associated with tumour initiation and survival. Besides, the TAMs polarization towards M2 phenotype is regulated by HCK activation, while pharmacological inhibition of HCK using RK20449 declined M2-like macrophage accumulation [134]. The phenomenon is consistent with previous finding that HCK primarily mediated M2-like immunosuppressive gene expression in macrophages/monocytes [135]. The emergence of this tyrosine kinase for macrophage polarization supports the potential use of SFK-inhibitor such as dasatinib and imatinib, to reprogram the immunosuppressive macrophages to anti-tumour phenotype through blunting the HCK activity of TAMs.

### Others

Recruitment of mast cells to tumour microenvironment has been shown to promote pancreatic cancer cell survival and inhibition of mast cell degranulation led to cancer cell death. Intervention of PCI-32765, the BTK inhibitor, declined mast cells degranulation and cancer cell growth [136]. The same phenomenon also documented in ibrutinib-treated pancreatic tumour, whereby ibrutinib treatment diminished the mast cell associated fibrosis [137]. Despite the effect of TKIs on neutrophils rarely been described, Patnaik et al. showed cabozantinib, multi-kinase inhibitor treatment induced neutrophils infiltration into prostate tumour sites. Further investigation postulated that dying tumour cells produced CXCL12 and HMGB1 to recruit and activate neutrophils for following cancer eradication after cabozantinib intervention [138].

## Targeting the non-cellular components

Extracellular matrix ECM components including fibronectin, laminin and collagen have been shown to determine the fate of cancer cells whether to remain dormant or proliferative [139]. It was demonstrated that collagen or fibronectin accumulation promoted progression of dormant breast cancer cells via β1-integrin mediated SRC and FAK activation. SFK inhibition with AZD0530 offered transient blockade on proliferation of dormant cancer cells, and only combination of AZD0530 with selumetinib, MEK inhibitor prevented the dormant-to-proliferative shift of cancer cells [140]. Furthermore, the composition of ECM also governs the therapeutic outcome of cancer treatment. Earlier study postulated that small cell lung cancer cells tend to accumulate at ECM rich region, whereby the ECM proteins further protected the cancer cells from chemotherapy. The adhesion of cancer cells to fibronectin triggered activation of protein tyrosine kinase and intervention with tyrphostin-25, a competitive EGFR tyrosine kinase inhibitor abolished the effect [141]. The similar phenomenon has also been documented by another two potent TKIs genistein and tyrophostin AG-1478 [142].

## Perspectives on combination treatment of TKIs and immunotherapy

It has been proposed in various types of human cancers that immunotherapeutic regimen could offer favourable outcome, though large-scale randomized, placebo-controlled trials shall be carried out prior to its application. Given the accumulating reports on acquired resistance of TKIs, the use of TKIs alone as first-line treatment seems challenging in spite of its better prognosis. TKIs treatment often gain rapid but not durable tumour response, which result in early improvement of survival curve of cancer patients without clear beneficial effect on the overall survival. The effect of TKIs could be strategically complemented by immunotherapy, which induces a low percentage but highly durable tumour response [143]. This notion has been partially supported by pre-clinical observation in mouse model, which showed that an ALK vaccine improved tumour relapses after TKI suspension [144]. The idea combining TKIs with immunotherapy raised in early 2010s and some efforts, in both pre-clinical and clinical extent, have been made to investigate its beneficial effect in cancer treatment [145]. Although most of the studies are still on-going, progress report in recent conference proceedings have suggested the prominent potential of this brilliant combination. For instance, McDermott et al. reported that combination of Atezolizumab (anti-PD-L1) with sunitinib (VEGFR inhibitor) resulted in encouraging anti-tumour effect than TKIs alone in metastatic renal cell carcinoma (mRCC), and a large scale phase III trial is highly suggested [146]. On-going studies from Ribas group suggested combination of pembrolizumab (anti-PD1) with dabrafenib (BRAF inhibitor) and trametinib (MEK inhibitor) for phase II study in BRAF-mutant melanoma patients after manageable toxic profile of this combination has been observed [147]. Mechanistically, the action of combination treatment may be in sequential or parallel. For instances, sequential use of sorafenib followed by sunitinb improved overall survival of the metastatic renal cell carcinoma patients, suggesting the sequential action of this combination [148]. However, given the regulation of TKIs on tumour microenvironment as we recognized in this review, it has been expected that the combination treatment would deliver a parallel action, that is, TKIs and immunotherapy synergize each other. It was observed that combination of anti-angiogenic TKIs sorafenib/sunitinib with anti-tumour rMVA–CEA–TRICOM vaccine, could synergistically increase the CD8+ tumour infiltrating lymphocytes and intratumoral CD11b + cells, which is hardly observed in TKIs or vaccine treatment alone. This is partially related to the regulation of TKIs alone on the phenotypes of immunosuppressive MDSCs and TAMs [149]. Combination of TKIs with FLT3-directed immunotherapy enhanced its therapeutic outcome on acute myeloid leukaemia, which is probably due to the induced expression and localization of FLT3 on the cell surface [150]. Efficacy and mechanism of action of combination treatment using TKIs and immunotherapy are expected to be under more extensive inspection.

Furthermore, safety of the combination use is still contradicting and unclear. Some studies have reported the increased risk of adverse reaction after combination therapy, including higher incidence of grade 3/4 liver enzyme elevation (40–70%) and high incidence of interstitial lung disease (38%) [151]. A lot of recent efforts were thus made to justify the safety use of combination treatment. A phase I study on the safety of combination treatment of cobimetinib (MEK inhibitor) and atezolizumab (anti-PD1) showed that the well tolerance of colorectal cancer patients at the maximum administered doses [152]. Combination of atezolizumab (anti-PD1) with vemurafenib (BRAF inhibitor) and cobimetinib (MEK inhibitor) induced rash and elevated liver enzymes in patents with BRAF-mutant melanoma, however, the adverse reaction was manageable by a run-in period of vemurafenib and cobimetinib [153]. However, unsuitability of combination treatment between immunotherapy and TKIs was also reported. For instances, combination of pazopanib (TKIs) and pembrolizumab (anti-PD1) resulted in significant hepatotoxicity in advanced RCC patients. While sequential regimen showed improved efficacy yet limited tolerability [154]. Interestingly, it was somewhat suggested that combination treatment may improve the adverse reaction, which may be probably due to the dose-reduction effect in TKIs. Bhatia et al. reported a favoured outcome in clinical trials using combination of IL21 with sorafenib to treat mRCC. The combination use of IL21 resulted in appropriate dose-reduction in sorafenib associated with improved adverse reaction [155]. These pilot findings suggested the safety of combinatory treatment of immunotherapy and TKIs on cancer patients warrant continued exploration for clinical benefit.

## Overcoming TKIs resistance through modulating tumor microenvironment

The development of acquired resistance remain as the major clinical challenge to TKIs treatment, despite TKIs are still the first line therapeutic regimen for many malignancies. For instances, non-small cell lung cancer patients who initially well responded to EGFR inhibitors, erlotinib or gefitinib had host adaptive response and tumor growth within 6–12 months [156, 157]. Majority of the first line TKIs aiming at the intrinsic function of tumor cells, while neglecting that the susceptibility of tumor cells may shift in response to the alteration of microenvironment. In fact, upon TKIs perturbation, the tumor microenvironment compartment tends to secrete the extrinsic factors such as cytokines, hormones, or growth factors in sensitizing or de-sensitizing the response of cancer cells. This notion was further supported by pre-clinical observations that the paracrine secretions of various growth factors by neighboring fibroblast such as hepatocyte growth factors (HGF) and neuregulin 1 (NRG1) further promoted cancer cell resistance to TKIs [60, 158]. While re-sensitization of cancer cells to TKIs is often achieved by either suppressing the secretive factors by TME or antagonizing the receptors on cancer cells. It was observed that co-administration of EGFR inhibitors with apatinib, a highly selective VEGFR2 inhibitor reduced the expression of VEGF in TME, which further delayed tumor growth in mice and prolonged disease-free survival in cancer patients [159]. The fibroblast and endothelial cells produced HGF and EGFR ligands reduced the cancer cell response to crizotinib, a dual inhibitor of ALK and Met. While co-administration of EGFR inhibitors enhanced the susceptibility of cancer cells to crizotinib [61]. These attempts suggest that manipulating TME by repressing pro-tumoral secretive factors or blocking receptors on cancer cells may be beneficial in overcoming TKIs resistance.

Also, it is believed that cancer cells develop an immunosuppressive microenvironment for evasion from immune surveillance upon TKIs intervention. Although the detailed mechanisms of acquired immune evasion is not yet understood, previous studies suggested that the inducible expression of immune checkpoints PD-1/PD-L1 may be involved. Notably, the elevated expression of PD-L1 was observed in EGFR-resistant tumor biopsy compared to pre-treated tumor samples. This suggests the activation of immune checkpoint may hinder the treatment efficacy of TKIs to cancer patients [160]. Furthermore, co-treatment of immune checkpoint inhibitor, nivolumab in lung cancer patients after disease progression with TKIs showed improved progression-free survival and the enhanced progression-free survival rate was in proportion to PD-L1 expression [161]. Similar observation was obtained from patients receiving ALK inhibitors, whereby anti-PD-1 immunotherapy restored the efficacy of ALK-targeted therapy [144]. Therefore, the treatment strategies aim at normalizing or reprogramming the immunosuppressive cancer environment may be promising in reversing TKIs resistance and achieve a favorable outcome of TKIs-associated cancer therapy.

## Our experience: Immuno-modulatory complementary therapy potentiates TKIs treatment

With recognition of the actions of TKIs on TME, our previous studies have also suggested the beneficial role of complementing TKIs with immune-modulatory therapy. Complementary treatment using PHY906, a composite herbal formula that had potential to increase the therapeutic index of cancer treatments in multiple clinical trials, was recently identified to modulate TME in sorafenib-treated hepatocellular carcinoma (HCC). This immune-modulatory effect of PHY906 improved the anti-tumour TME signature and thereby enhanced the efficacy of sorafenib [162]. An active component isolated from PHY906, named baicalin, has evidenced to contribute to this immuno-modulatory action by specifically targeting on autophagic pathway in TAMs [163]. These observations combined with our study on other natural molecules suggest the potential of using immuno-modulatory complementary therapy [164] in combination with TKIs as emerging cancer therapy.

## Conclusion

In past decade, tyrosine kinase inhibitors targeting at tumour cells represent a promising therapeutic agent for cancer patients; yet, the use of TKIs in modulating tumour microenvironment is still in its infancy. The recent advances we summarized here emphasize the potential use of TKIs for re-educating or normalizing the dysregulated tumour microenvironment for cancer treatment. An understanding of the modulatory effect of TKIs on tumour microenvironment could expedite the maximal use of TKIs on cancer therapy as well as re-positions the existing TKIs as combination cancer treatment. Furthermore, phosphorylated status of target protein could also be determined by protein phosphatase, which belongs to another group of protein involved in microenvironment status of tumour cells, is worthy for further exploration. Much more efforts, either pre-clinically or clinically, is therefore anticipated in order to maximize the efficacy and safety for patients.

## References

[1] Khoury GA, Baliban RC, Floudas CA. Proteome-wide post-translational modification statistics: frequency analysis and curation of the swiss-prot database. Sci Rep 2011;1:90. 10.1038/srep00090 PMC3201773 22034591

[2] Manning G, Whyte DB, Martinez R, Hunter T, Sudarsanam S (2002). The protein Kinase complement of the human genome. Science.

[3] Robinson DR, Wu YM, Lin SF (2000). The protein tyrosine kinase family of the human genome. Oncogene.

[4] Paul MK, Mukhopadhyay AK (2004). Tyrosine kinase – role and significance in cancer. Int J Med Sci.

[5] Sangwan V, Park M (2006). Receptor tyrosine kinases: role in cancer progression. Curr Oncol.

[6] Heldin CH (2013). Targeting the PDGF signaling pathway in tumor treatment. Cell Commun Signal.

[7] Normanno N, De Luca A, Bianco C, Strizzi L, Mancino M, Maiello MR, Carotenuto A, De Feo G, Caponigro F, Salomon DS (2006). Epidermal growth factor receptor (EGFR) signaling in cancer. Gene.

[8] Noble MEM, Endicott JA, Johnson LN (2004). Protein Kinase inhibitors: insights into drug design from structure. Science.

[9] Yang JJ, Zhou Q, Yan HH, Zhang XC, Chen HJ, Tu HY, Wang Z, Xu CR, Su J, Wang BC (2017). A phase III randomised controlled trial of erlotinib vs gefitinib in advanced non-small cell lung cancer with EGFR mutations. Br J Cancer.

[10] Hochhaus A, Larson RA, Guilhot F, Radich JP, Branford S, Hughes TP, Baccarani M, Deininger MW, Cervantes F, Fujihara S (2017). Long-term outcomes of Imatinib treatment for chronic myeloid leukemia. N Engl J Med.

[11] Casali PG, Zalcberg J, Le Cesne A, Reichardt P, Blay JY, Lindner LH, Judson IR, Schoffski P, Leyvraz S, Italiano A (2017). Ten-year progression-free and overall survival in patients with Unresectable or metastatic GI Stromal tumors: long-term analysis of the European Organisation for Research and Treatment of Cancer, Italian sarcoma group, and Australasian gastrointestinal trials group intergroup phase III randomized trial on Imatinib at two dose levels. J Clin Oncol.

[12] Haas NB, Manola J, Dutcher JP, Flaherty KT, Uzzo RG, Atkins MB, DiPaola RS, Choueiri TK (2017). Adjuvant treatment for high-risk clear cell renal cancer: updated results of a high-risk subset of the ASSURE randomized trial. JAMA Oncol.

[13] Bruix J, Takayama T, Mazzaferro V, Chau GY, Yang J, Kudo M, Cai J, Poon RT, Han KH, Tak WY (2015). Adjuvant sorafenib for hepatocellular carcinoma after resection or ablation (STORM): a phase 3, randomised, double-blind, placebo-controlled trial. Lancet Oncol.

[14] Gross S, Rahal R, Stransky N, Lengauer C, Hoeflich KP (2015). Targeting cancer with kinase inhibitors. J Clin Invest.

[15] Chen F, Zhuang X, Lin L, Yu P, Wang Y, Shi Y, Hu G, Sun Y (2015). New horizons in tumor microenvironment biology: challenges and opportunities. BMC Med.

[16] Yao Z, Fenoglio S, Gao DC, Camiolo M, Stiles B, Lindsted T, Schlederer M, Johns C, Altorki N, Mittal V (2010). TGF-beta IL-6 axis mediates selective and adaptive mechanisms of resistance to molecular targeted therapy in lung cancer. Proc Natl Acad Sci U S A.

[17] Kaiko GE, Horvat JC, Beagley KW, Hansbro PM (2008). Immunological decision-making: how does the immune system decide to mount a helper T-cell response?. Immunology.

[18] Kalluri R, Zeisberg M (2006). Fibroblasts in cancer. Nat Rev Cancer.

[19] Kwilas AR, Donahue RN, Tsang KY, Hodge JW. Immune consequences of tyrosine kinase inhibitors that synergize with cancer immunotherapy. Cancer Cell Microenviron 2015;2:e677. 10.14800/ccm.677 PMC4440700 26005708

[20] Baker CH, Kedar D, McCarty MF, Tsan R, Weber KL, Bucana CD, Fidler IJ (2002). Blockade of epidermal growth factor receptor signaling on tumor cells and tumor-associated endothelial cells for therapy of human carcinomas. Am J Pathol.

[21] Cerniglia GJ, Pore N, Tsai JH, Schultz S, Mick R, Choe R, Xing X, Durduran T, Yodh AG, Evans SM (2009). Epidermal growth factor receptor inhibition modulates the microenvironment by vascular normalization to improve chemotherapy and radiotherapy efficacy. PLoS One.

[22] Moasser MM, Wilmes LJ, Wong CH, Aliu S, Li KL, Wang D, Hom YK, Hann B, Hylton NM (2007). Improved tumor vascular function following high-dose epidermal growth factor receptor tyrosine kinase inhibitor therapy. J Magn Reson Imaging.

[23] Philippova M, Pfaff D, Kyriakakis E, Buechner SA, Iezzi G, Spagnoli GC, Schoenenberger AW, Erne P, Resink TJ (2013). T-cadherin loss promotes experimental metastasis of squamous cell carcinoma. Eur J Cancer.

[24] Iivanainen E, Lauttia S, Zhang N, Tvorogov D, Kulmala J, Grenman R, Salven P, Elenius K (2009). The EGFR inhibitor gefitinib suppresses recruitment of pericytes and bone marrow-derived perivascular cells into tumor vessels. Microvasc Res.

[25] Shibuya M (2011). Vascular endothelial growth factor (VEGF) and its receptor (VEGFR) signaling in angiogenesis: a crucial target for anti- and pro-Angiogenic therapies. Genes Cancer.

[26] Geng L, Donnelly E, McMahon G, Lin PC, Sierra-Rivera E, Oshinka H, Hallahan DE (2001). Inhibition of vascular endothelial growth factor receptor signaling leads to reversal of tumor resistance to radiotherapy. Cancer Res.

[27] Mathew P, Fidler IJ, Logothetis CJ (2004). Combination docetaxel and platelet-derived growth factor receptor inhibition with imatinib mesylate in prostate cancer. Semin Oncol.

[28] Akiyama K, Ohga N, Hida Y, Kawamoto T, Sadamoto Y, Ishikawa S, Maishi N, Akino T, Kondoh M, Matsuda A (2012). Tumor endothelial cells acquire drug resistance by MDR1 up-regulation via VEGF signaling in tumor microenvironment. Am J Pathol.

[29] Yazici S, Kim SJ, Busby JE, He J, Thaker P, Yokoi K, Fan D, Fidler IJ (2005). Dual inhibition of the epidermal growth factor and vascular endothelial growth factor phosphorylation for antivascular therapy of human prostate cancer in the prostate of nude mice. Prostate.

[30] Drusbosky L, Gars E, Trujillo A, McGee C, Meacham A, Wise E, Scott EW, Cogle CR (2015). Endothelial cell derived angiocrine support of acute myeloid leukemia targeted by receptor tyrosine kinase inhibition. Leuk Res.

[31] Blansfield JA, Caragacianu D, Alexander HR, Tangrea MA, Morita SY, Lorang D, Schafer P, Muller G, Stirling D, Royal RE, Libutti SK (2008). Combining agents that target the tumor microenvironment improves the efficacy of anticancer therapy. Clin Cancer Res.

[32] Hatipoglu G, Hock SW, Weiss R, Fan Z, Sehm T, Ghoochani A, Buchfelder M, Savaskan NE, Eyupoglu IY (2015). Sunitinib impedes brain tumor progression and reduces tumor-induced neurodegeneration in the microenvironment. Cancer Sci.

[33] Coluccia AM, Cirulli T, Neri P, Mangieri D, Colanardi MC, Gnoni A, Di Renzo N, Dammacco F, Tassone P, Ribatti D (2008). Validation of PDGFRbeta and c-Src tyrosine kinases as tumor/vessel targets in patients with multiple myeloma: preclinical efficacy of the novel, orally available inhibitor dasatinib. Blood.

[34] Cooke VG, LeBleu VS, Keskin D, Khan Z, O'Connell JT, Teng Y, Duncan MB, Xie L, Maeda G, Vong S (2012). Pericyte depletion results in hypoxia-associated epithelial-to-mesenchymal transition and metastasis mediated by met signaling pathway. Cancer Cell.

[35] Kim SJ, Uehara H, Yazici S, He J, Langley RR, Mathew P, Fan D, Fidler IJ (2005). Modulation of bone microenvironment with zoledronate enhances the therapeutic effects of STI571 and paclitaxel against experimental bone metastasis of human prostate cancer. Cancer Res.

[36] Andersen JB, Spee B, Blechacz BR, Avital I, Komuta M, Barbour A, Conner EA, Gillen MC, Roskams T, Roberts LR (2012). Genomic and genetic characterization of cholangiocarcinoma identifies therapeutic targets for tyrosine kinase inhibitors. Gastroenterology.

[37] Bhowmick NA, Neilson EG, Moses HL (2004). Stromal fibroblasts in cancer initiation and progression. Nature.

[38] Grinnell F (2003). Fibroblast biology in three-dimensional collagen matrices. Trends Cell Biol.

[39] Zhang B, Li M, McDonald T, Holyoake TL, Moon RT, Campana D, Shultz L, Bhatia R (2013). Microenvironmental protection of CML stem and progenitor cells from tyrosine kinase inhibitors through N-cadherin and Wnt-beta-catenin signaling. Blood.

[40] Piechocki MP (2008). A stable explant culture of HER2/neu invasive carcinoma supported by alpha-smooth muscle Actin expressing stromal cells to evaluate therapeutic agents. BMC Cancer.

[41] Cornall RJ, Cheng AM, Pawson T, Goodnow CC (2000). Role of Syk in B-cell development and antigen-receptor signaling. Proc Natl Acad Sci U S A.

[42] Buchner M, Baer C, Prinz G, Dierks C, Burger M, Zenz T, Stilgenbauer S, Jumaa H, Veelken H, Zirlik K (2010). Spleen tyrosine kinase inhibition prevents chemokine- and integrin-mediated stromal protective effects in chronic lymphocytic leukemia. Blood.

[43] Stokes JB, Adair SJ, Slack-Davis JK, Walters DM, Tilghman RW, Hershey ED, Lowrey B, Thomas KS, Bouton AH, Hwang RF (2011). Inhibition of focal adhesion kinase by PF-562,271 inhibits the growth and metastasis of pancreatic cancer concomitant with altering the tumor microenvironment. Mol Cancer Ther.

[44] Takigawa H, Kitadai Y, Shinagawa K, Yuge R, Higashi Y, Tanaka S, Yasui W, Chayama K (2016). Multikinase inhibitor regorafenib inhibits the growth and metastasis of colon cancer with abundant stroma. Cancer Sci.

[45] Albrengues J, Bourget I, Pons C, Butet V, Hofman P, Tartare-Deckert S, Feral CC, Meneguzzi G, Gaggioli C (2014). LIF mediates proinvasive activation of stromal fibroblasts in cancer. Cell Rep.

[46] Quintarelli C, De Angelis B, Errichiello S, Caruso S, Esposito N, Colavita I, Raia M, Pagliuca S, Pugliese N, Risitano AM (2014). Selective strong synergism of Ruxolitinib and second generation tyrosine kinase inhibitors to overcome bone marrow stroma related drug resistance in chronic myelogenous leukemia. Leuk Res.

[47] Pietras K, Pahler J, Bergers G, Hanahan D (2008). Functions of paracrine PDGF signaling in the proangiogenic tumor stroma revealed by pharmacological targeting. PLoS Med.

[48] Yuge R, Kitadai Y, Shinagawa K, Onoyama M, Tanaka S, Yasui W, Chayama K (2015). mTOR and PDGF pathway blockade inhibits liver metastasis of colorectal cancer by modulating the tumor microenvironment. Am J Pathol.

[49] Catena R, Luis-Ravelo D, Anton I, Zandueta C, Salazar-Colocho P, Larzabal L, Calvo A, Lecanda F (2011). PDGFR signaling blockade in marrow stroma impairs lung cancer bone metastasis. Cancer Res.

[50] Wan X, Corn PG, Yang J, Palanisamy N, Starbuck MW, Efstathiou E, Li Ning Tapia EM, Zurita AJ, Aparicio A, Ravoori MK (2014). Prostate cancer cell-stromal cell crosstalk via FGFR1 mediates antitumor activity of dovitinib in bone metastases. Sci Transl Med.

[51] Wang ZH, Li Q, Ruan SQ, Xiao Q, Liu Y, Hu YT, Hu LF, Chen HY, Zheng S, Zhang SZ, Ding KF (2014). Sunitinib mesylate inhibits proliferation of human colonic stromal fibroblasts in vitro and in vivo. J Zhejiang Univ Sci B.

[52] Traer E, Javidi-Sharifi N, Agarwal A, Dunlap J, English I, Martinez J, Tyner JW, Wong M, Druker BJ (2014). Ponatinib overcomes FGF2-mediated resistance in CML patients without kinase domain mutations. Blood.

[53] Saito S, Morishima K, Ui T, Hoshino H, Matsubara D, Ishikawa S, Aburatani H, Fukayama M, Hosoya Y, Sata N (2015). The role of HGF/MET and FGF/FGFR in fibroblast-derived growth stimulation and lapatinib-resistance of esophageal squamous cell carcinoma. BMC Cancer.

[54] Yoshida T, Ishii G, Goto K, Neri S, Hashimoto H, Yoh K, Niho S, Umemura S, Matsumoto S, Ohmatsu H (2015). Podoplanin-positive cancer-associated fibroblasts in the tumor microenvironment induce primary resistance to EGFR-TKIs in lung adenocarcinoma with EGFR mutation. Clin Cancer Res.

[55] Blumenschein GR, Mills GB, Gonzalez-Angulo AM (2012). Targeting the Hepatocyte growth factor–cMET Axis in cancer therapy. J Clin Oncol.

[56] Mueller KL, Madden JM, Zoratti GL, Kuperwasser C, List K, Boerner JL (2012). Fibroblast-secreted hepatocyte growth factor mediates epidermal growth factor receptor tyrosine kinase inhibitor resistance in triple-negative breast cancers through paracrine activation of met. Breast Cancer Res.

[57] Yano S, Yamada T, Takeuchi S, Tachibana K, Minami Y, Yatabe Y, Mitsudomi T, Tanaka H, Kimura T, Kudoh S (2011). Hepatocyte growth factor expression in EGFR mutant lung cancer with intrinsic and acquired resistance to tyrosine kinase inhibitors in a Japanese cohort. J Thorac Oncol.

[58] Straussman R, Morikawa T, Shee K, Barzily-Rokni M, Qian ZR, Du J, Davis A, Mongare MM, Gould J, Frederick DT (2012). Tumour micro-environment elicits innate resistance to RAF inhibitors through HGF secretion. Nature.

[59] Li M, Xin X, Wu T, Hua T, Wang H, Wang H (2015). Stromal cells of endometrial carcinoma promotes proliferation of epithelial cells through the HGF/c-met/Akt signaling pathway. Tumour Biol.

[60] Wang W, Li Q, Yamada T, Matsumoto K, Matsumoto I, Oda M, Watanabe G, Kayano Y, Nishioka Y, Sone S, Yano S (2009). Crosstalk to stromal fibroblasts induces resistance of lung cancer to epidermal growth factor receptor tyrosine kinase inhibitors. Clin Cancer Res.

[61] Yamada T, Takeuchi S, Nakade J, Kita K, Nakagawa T, Nanjo S, Nakamura T, Matsumoto K, Soda M, Mano H (2012). Paracrine receptor activation by microenvironment triggers bypass survival signals and ALK inhibitor resistance in EML4-ALK lung cancer cells. Clin Cancer Res.

[62] Kwon Y, Smith BD, Zhou Y, Kaufman MD, Godwin AK (2015). Effective inhibition of c-MET-mediated signaling, growth and migration of ovarian cancer cells is influenced by the ovarian tissue microenvironment. Oncogene.

[63] Hirata E, Girotti MR, Viros A, Hooper S, Spencer-Dene B, Matsuda M, Larkin J, Marais R, Sahai E (2015). Intravital imaging reveals how BRAF inhibition generates drug-tolerant microenvironments with high integrin beta1/FAK signaling. Cancer Cell.

[64] Yan XL, Fu CJ, Chen L, Qin JH, Zeng Q, Yuan HF, Nan X, Chen HX, Zhou JN, Lin YL (2012). Mesenchymal stem cells from primary breast cancer tissue promote cancer proliferation and enhance mammosphere formation partially via EGF/EGFR/Akt pathway. Breast Cancer Res Treat.

[65] Yoshida GJ, Saya H (2014). EpCAM expression in the prostate cancer makes the difference in the response to growth factors. Biochem Biophys Res Commun.

[66] Lacerda L, Debeb BG, Smith D, Larson R, Solley T, Xu W, Krishnamurthy S, Gong Y, Levy LB, Buchholz T (2015). Mesenchymal stem cells mediate the clinical phenotype of inflammatory breast cancer in a preclinical model. Breast Cancer Res.

[67] Draghiciu O, Nijman HW, Hoogeboom BN, Meijerhof T, Daemen T (2015). Sunitinib depletes myeloid-derived suppressor cells and synergizes with a cancer vaccine to enhance antigen-specific immune responses and tumor eradication. Oncoimmunology.

[68] Kitayama J, Emoto S, Yamaguchi H, Ishigami H, Watanabe T (2014). CD90+ mesothelial-like cells in peritoneal fluid promote peritoneal metastasis by forming a tumor permissive microenvironment. PLoS One.

[69] Zhang B, Ho YW, Huang Q, Maeda T, Lin A, Lee SU, Hair A, Holyoake TL, Huettner C, Bhatia R (2012). Altered microenvironmental regulation of leukemic and normal stem cells in chronic myelogenous leukemia. Cancer Cell.

[70] Goldman A, Majumder B, Dhawan A, Ravi S, Goldman D, Kohandel M, Majumder PK, Sengupta S (2015). Temporally sequenced anticancer drugs overcome adaptive resistance by targeting a vulnerable chemotherapy-induced phenotypic transition. Nat Commun.

[71] Pohorelic B, Singh R, Parkin S, Koro K, Yang AD, Egan C, Magliocco A (2012). Role of Src in breast cancer cell migration and invasion in a breast cell/bone-derived cell microenvironment. Breast Cancer Res Treat.

[72] Ono K, Kamiya S, Akatsu T, Nakamura C, Li M, Amizuka N, Matsumoto K, Nakamura T, Kugai N, Wada S (2006). Involvement of hepatocyte growth factor in the development of bone metastasis of a mouse mammary cancer cell line, BALB/c-MC. Bone.

[73] Brama M, Basciani S, Cherubini S, Mariani S, Migliaccio S, Arizzi M, Rosano G, Spera G, Gnessi L (2007). Osteoblast-conditioned medium promotes proliferation and sensitizes breast cancer cells to imatinib treatment. Endocr Relat Cancer.

[74] Buettner R, Mesa T, Vultur A, Lee F, Jove R (2008). Inhibition of Src family kinases with dasatinib blocks migration and invasion of human melanoma cells. Mol Cancer Res.

[75] Liang W, Kujawski M, Wu J, Lu J, Herrmann A, Loera S, Yen Y, Lee F, Yu H, Wen W, Jove R (2010). Antitumor activity of targeting SRC kinases in endothelial and myeloid cell compartments of the tumor microenvironment. Clin Cancer Res.

[76] Xin H, Herrmann A, Reckamp K, Zhang W, Pal S, Hedvat M, Zhang C, Liang W, Scuto A, Weng S (2011). Antiangiogenic and antimetastatic activity of JAK inhibitor AZD1480. Cancer Res.

[77] Alizadeh D, Larmonier N (2014). Chemotherapeutic targeting of cancer-induced immunosuppressive cells. Cancer Res.

[78] Draghiciu O, Lubbers J, Nijman HW, Daemen T (2015). Myeloid derived suppressor cells-an overview of combat strategies to increase immunotherapy efficacy. Oncoimmunology.

[79] Ozao-Choy J, Ma G, Kao J, Wang GX, Meseck M, Sung M, Schwartz M, Divino CM, Pan PY, Chen SH (2009). The novel role of tyrosine kinase inhibitor in the reversal of immune suppression and modulation of tumor microenvironment for immune-based cancer therapies. Cancer Res.

[80] Xin H, Zhang C, Herrmann A, Du Y, Figlin R, Yu H (2009). Sunitinib inhibition of Stat3 induces renal cell carcinoma tumor cell apoptosis and reduces immunosuppressive cells. Cancer Res.

[81] Zhang X, Fang X, Gao Z, Chen W, Tao F, Cai P, Yuan H, Shu Y, Xu Q, Sun Y, Gu Y (2014). Axitinib, a selective inhibitor of vascular endothelial growth factor receptor, exerts an anticancer effect in melanoma through promoting antitumor immunity. Anti-Cancer Drugs.

[82] Bose A, Lowe DB, Rao A, Storkus WJ (2012). Combined vaccine+axitinib therapy yields superior antitumor efficacy in a murine melanoma model. Melanoma Res.

[83] Kwilas AR, Ardiani A, Donahue RN, Aftab DT, Hodge JW (2014). Dual effects of a targeted small-molecule inhibitor (cabozantinib) on immune-mediated killing of tumor cells and immune tumor microenvironment permissiveness when combined with a cancer vaccine. J Transl Med.

[84] Yu N, Fu S, Xu Z, Liu Y, Hao J, Zhang A, Wang B (2016). Synergistic antitumor responses by combined GITR activation and sunitinib in metastatic renal cell carcinoma. Int J Cancer.

[85] Huo M, Zhao Y, Satterlee AB, Wang Y, Xu Y, Huang L (2017). Tumor-targeted delivery of sunitinib base enhances vaccine therapy for advanced melanoma by remodeling the tumor microenvironment. J Control Release.

[86] Ko JS, Rayman P, Ireland J, Swaidani S, Li G, Bunting KD, Rini B, Finke JH, Cohen PA (2010). Direct and differential suppression of myeloid-derived suppressor cell subsets by sunitinib is compartmentally constrained. Cancer Res.

[87] Finke J, Ko J, Rini B, Rayman P, Ireland J, Cohen P (2011). MDSC as a mechanism of tumor escape from sunitinib mediated anti-angiogenic therapy. Int Immunopharmacol.

[88] Piao Y, Liang J, Holmes L, Zurita AJ, Henry V, Heymach JV, de Groot JF (2012). Glioblastoma resistance to anti-VEGF therapy is associated with myeloid cell infiltration, stem cell accumulation, and a mesenchymal phenotype. Neuro-Oncology.

[89] Liu L, Ye TH, Han YP, Song H, Zhang YK, Xia Y, Wang NY, Xiong Y, Song XJ, Zhu YX (2014). Reductions in myeloid-derived suppressor cells and lung metastases using AZD4547 treatment of a metastatic murine breast tumor model. Cell Physiol Biochem.

[90] Holdman XB, Welte T, Rajapakshe K, Pond A, Coarfa C, Mo Q, Huang S, Hilsenbeck SG, Edwards DP, Zhang X, Rosen JM (2015). Upregulation of EGFR signaling is correlated with tumor stroma remodeling and tumor recurrence in FGFR1-driven breast cancer. Breast Cancer Res.

[91] Steinberg SM, Shabaneh TB, Zhang P, Martyanov V, Li Z, Malik BT, Wood TA, Boni A, Molodtsov A, Angeles CV (2017). Myeloid cells that impair immunotherapy are restored in melanomas with acquired resistance to BRAF inhibitors. Cancer Res.

[92] Lindau D, Gielen P, Kroesen M, Wesseling P, Adema GJ (2013). The immunosuppressive tumour network: myeloid-derived suppressor cells, regulatory T cells and natural killer T cells. Immunology.

[93] Kujawski M, Zhang C, Herrmann A, Reckamp K, Scuto A, Jensen M, Deng J, Forman S, Figlin R, Yu H (2010). Targeting STAT3 in adoptively transferred T cells promotes their in vivo expansion and antitumor effects. Cancer Res.

[94] Hendriks RW, Yuvaraj S, Kil LP (2014). Targeting Bruton's tyrosine kinase in B cell malignancies. Nat Rev Cancer.

[95] Niemann CU, Herman SE, Maric I, Gomez-Rodriguez J, Biancotto A, Chang BY, Martyr S, Stetler-Stevenson M, Yuan CM, Calvo KR (2016). Disruption of in vivo chronic lymphocytic leukemia tumor-microenvironment interactions by Ibrutinib--findings from an investigator-initiated phase II study. Clin Cancer Res.

[96] Sagiv-Barfi I, Kohrt HE, Burckhardt L, Czerwinski DK, Levy R (2015). Ibrutinib enhances the antitumor immune response induced by intratumoral injection of a TLR9 ligand in mouse lymphoma. Blood.

[97] Gritsina G, Xiao F, O'Brien SW, Gabbasov R, Maglaty MA, Xu RH, Thapa RJ, Zhou Y, Nicolas E, Litwin S (2015). Targeted blockade of JAK/STAT3 signaling inhibits ovarian carcinoma growth. Mol Cancer Ther.

[98] Kobayashi A, Tanizaki Y, Kimura A, Ishida Y, Nosaka M, Toujima S, Kuninaka Y, Minami S, Ino K, Kondo T (2015). AG490, a Jak2 inhibitor, suppressed the progression of murine ovarian cancer. Eur J Pharmacol.

[99] Du Four S, Maenhout SK, Benteyn D, De Keersmaecker B, Duerinck J, Thielemans K, Neyns B, Aerts JL (2016). Disease progression in recurrent glioblastoma patients treated with the VEGFR inhibitor axitinib is associated with increased regulatory T cell numbers and T cell exhaustion. Cancer Immunol Immunother.

[100] Serrels A, Lund T, Serrels B, Byron A, McPherson RC, von Kriegsheim A, Gomez-Cuadrado L, Canel M, Muir M, Ring JE (2015). Nuclear FAK controls chemokine transcription, Tregs, and evasion of anti-tumor immunity. Cell.

[101] Jiang H, Hegde S. Targeting focal adhesion kinase renders pancreatic cancers responsive to checkpoint immunotherapy. Nat Med. 2016;22:851–60. 10.1038/nm.4123 PMC4935930 27376576

[102] Fei F, Yu Y, Schmitt A, Rojewski MT, Chen B, Gotz M, Dohner H, Bunjes D, Schmitt M (2009). Dasatinib inhibits the proliferation and function of CD4+CD25+ regulatory T cells. Br J Haematol.

[103] Fraser CK, Blake SJ, Diener KR, Lyons AB, Brown MP, Hughes TP, Hayball JD (2009). Dasatinib inhibits recombinant viral antigen-specific murine CD4+ and CD8+ T-cell responses and NK-cell cytolytic activity in vitro and in vivo. Exp Hematol.

[104] Yang Y, Liu C, Peng W, Lizee G, Overwijk WW, Liu Y, Woodman SE, Hwu P (2012). Antitumor T-cell responses contribute to the effects of dasatinib on c-KIT mutant murine mastocytoma and are potentiated by anti-OX40. Blood.

[105] Balachandran VP, Cavnar MJ, Zeng S, Bamboat ZM, Ocuin LM, Obaid H, Sorenson EC, Popow R, Ariyan C, Rossi F (2011). Imatinib potentiates antitumor T cell responses in gastrointestinal stromal tumor through the inhibition of Ido. Nat Med.

[106] Zhao W, Gu YH, Song R, Qu BQ, Xu Q (2008). Sorafenib inhibits activation of human peripheral blood T cells by targeting LCK phosphorylation. Leukemia.

[107] Houben R, Voigt H, Noelke C, Hofmeister V, Becker JC, Schrama D (2009). MAPK-independent impairment of T-cell responses by the multikinase inhibitor sorafenib. Mol Cancer Ther.

[108] Chen ML, Yan BS, Lu WC, Chen MH, Yu SL, Yang PC, Cheng AL (2014). Sorafenib relieves cell-intrinsic and cell-extrinsic inhibitions of effector T cells in tumor microenvironment to augment antitumor immunity. Int J Cancer.

[109] Sunay MM, Foote JB, Leatherman JM, Edwards JP, Armstrong TD, Nirschl CJ, Hicks J, Emens LA (2017). Sorafenib combined with HER-2 targeted vaccination can promote effective T cell immunity in vivo. Int Immunopharmacol.

[110] Nagai H, Mukozu T, Matsui D, Kanekawa T, Kanayama M, Wakui N, Momiyama K, Shinohara M, Iida K, Ishii K (2012). Sorafenib prevents escape from host immunity in liver cirrhosis patients with advanced hepatocellular carcinoma. Clin Dev Immunol.

[111] Chen Y, Ramjiawan RR, Reiberger T, Ng MR, Hato T, Huang Y, Ochiai H, Kitahara S, Unan EC, Reddy TP (2015). CXCR4 inhibition in tumor microenvironment facilitates anti-programmed death receptor-1 immunotherapy in sorafenib-treated hepatocellular carcinoma in mice. Hepatology.

[112] Cabrera R, Ararat M, Xu Y, Brusko T, Wasserfall C, Atkinson MA, Chang LJ, Liu C, Nelson DR (2013). Immune modulation of effector CD4+ and regulatory T cell function by sorafenib in patients with hepatocellular carcinoma. Cancer Immunol Immunother.

[113] Frederick DT, Piris A, Cogdill AP, Cooper ZA, Lezcano C, Ferrone CR, Mitra D, Boni A, Newton LP, Liu C (2013). BRAF inhibition is associated with enhanced melanoma antigen expression and a more favorable tumor microenvironment in patients with metastatic melanoma. Clin Cancer Res.

[114] Martinelli E, Morgillo F, Troiani T, Ciardiello F (2017). Cancer resistance to therapies against the EGFR-RAS-RAF pathway: the role of MEK. Cancer Treat Rev.

[115] Hume DA, MacDonald KP (2012). Therapeutic applications of macrophage colony-stimulating factor-1 (CSF-1) and antagonists of CSF-1 receptor (CSF-1R) signaling. Blood.

[116] Brownlow N, Mol C, Hayford C, Ghaem-Maghami S, Dibb NJ (2009). Dasatinib is a potent inhibitor of tumour-associated macrophages, osteoclasts and the FMS receptor. Leukemia.

[117] Dewar AL, Cambareri AC, Zannettino AC, Miller BL, Doherty KV, Hughes TP, Lyons AB (2005). Macrophage colony-stimulating factor receptor c-fms is a novel target of imatinib. Blood.

[118] Cavnar MJ, Zeng S, Kim TS, Sorenson EC, Ocuin LM, Balachandran VP, Seifert AM, Greer JB, Popow R, Crawley MH (2013). KIT oncogene inhibition drives intratumoral macrophage M2 polarization. J Exp Med.

[119] Zhang C, Ibrahim PN, Zhang J, Burton EA, Habets G, Zhang Y, Powell B, West BL, Matusow B, Tsang G (2013). Design and pharmacology of a highly specific dual FMS and KIT kinase inhibitor. Proc Natl Acad Sci U S A.

[120] DeNardo DG, Brennan DJ, Rexhepaj E, Ruffell B, Shiao SL, Madden SF, Gallagher WM, Wadhwani N, Keil SD, Junaid SA (2011). Leukocyte complexity predicts breast cancer survival and functionally regulates response to chemotherapy. Cancer Discov.

[121] Polk A, Lu Y, Wang T, Seymour E, Bailey NG, Singer JW, Boonstra PS, Lim MS, Malek S, Wilcox RA (2016). Colony-stimulating Factor-1 receptor is required for nurse-like cell survival in chronic lymphocytic leukemia. Clin Cancer Res.

[122] Sprinzl MF, Reisinger F, Puschnik A, Ringelhan M, Ackermann K, Hartmann D, Schiemann M, Weinmann A, Galle PR, Schuchmann M (2013). Sorafenib perpetuates cellular anticancer effector functions by modulating the crosstalk between macrophages and natural killer cells. Hepatology.

[123] De Palma M, Venneri MA, Galli R, Sergi LS, Politi LS, Sampaolesi M, Naldini L (2005). Tie2 identifies a hematopoietic lineage of proangiogenic monocytes required for tumor vessel formation and a mesenchymal population of pericyte progenitors. Cancer Cell.

[124] Nakazawa Y, Kawano S, Matsui J, Funahashi Y, Tohyama O, Muto H, Nakagawa T, Matsushima T (2015). Multitargeting strategy using lenvatinib and golvatinib: maximizing anti-angiogenesis activity in a preclinical cancer model. Cancer Sci.

[125] Goede V, Coutelle O, Neuneier J, Reinacher-Schick A, Schnell R, Koslowsky TC, Weihrauch MR, Cremer B, Kashkar H, Odenthal M (2010). Identification of serum angiopoietin-2 as a biomarker for clinical outcome of colorectal cancer patients treated with bevacizumab-containing therapy. Br J Cancer.

[126] Peterson TE, Kirkpatrick ND, Huang Y, Farrar CT, Marijt KA, Kloepper J, Datta M, Amoozgar Z, Seano G, Jung K (2016). Dual inhibition of Ang-2 and VEGF receptors normalizes tumor vasculature and prolongs survival in glioblastoma by altering macrophages. Proc Natl Acad Sci U S A.

[127] Scott RS, McMahon EJ, Pop SM, Reap EA, Caricchio R, Cohen PL, Earp HS, Matsushima GK (2001). Phagocytosis and clearance of apoptotic cells is mediated by MER. Nature.

[128] Wu J, Frady LN, Bash RE, Cohen SM, Schorzman AN, Su YT, Irvin DM, Zamboni WC, Wang X, Frye SV, et al. MerTK as a therapeutic target in glioblastoma. Neuro Oncol. 2017; 20:92-102.. 10.1093/neuonc/nox111 PMC5761530 28605477

[129] Stanford JC, Young C, Hicks D, Owens P, Williams A, Vaught DB, Morrison MM, Lim J, Williams M, Brantley-Sieders DM (2014). Efferocytosis produces a prometastatic landscape during postpartum mammary gland involution. J Clin Invest.

[130] Ní Gabhann J, Jefferies CA (2011). TLR-induced activation of Btk – role for endosomal MHC class II molecules revealed. Cell Res.

[131] Lee KG, Xu S, Kang ZH, Huo J, Huang M, Liu D, Takeuchi O, Akira S, Lam KP (2012). Bruton's tyrosine kinase phosphorylates toll-like receptor 3 to initiate antiviral response. Proc Natl Acad Sci U S A.

[132] Ping L, Ding N, Shi Y, Feng L, Li J, Liu Y, Lin Y, Shi C, Wang X, Pan Z (2017). The Bruton's tyrosine kinase inhibitor ibrutinib exerts immunomodulatory effects through regulation of tumor-infiltrating macrophages. Oncotarget.

[133] Fiorcari S, Maffei R, Audrito V, Martinelli S, Ten Hacken E, Zucchini P, Grisendi G, Potenza L, Luppi M, Burger JA (2016). Ibrutinib modifies the function of monocyte/macrophage population in chronic lymphocytic leukemia. Oncotarget.

[134] Poh AR, Love CG, Masson F, Preaudet A, Tsui C, Whitehead L, Monard S, Khakham Y, Burstroem L, Lessene G (2017). Inhibition of hematopoietic cell Kinase activity suppresses myeloid cell-mediated colon cancer progression. Cancer Cell.

[135] Bhattacharjee A, Pal S, Feldman GM, Cathcart MK (2011). Hck is a key regulator of gene expression in alternatively activated human monocytes. J Biol Chem.

[136] Soucek L, Buggy JJ, Kortlever R, Adimoolam S, Monclus HA, Allende MT, Swigart LB, Evan GI (2011). Modeling pharmacological inhibition of mast cell degranulation as a therapy for insulinoma. Neoplasia.

[137] Masso-Valles D, Jauset T, Serrano E, Sodir NM, Pedersen K, Affara NI, Whitfield JR, Beaulieu ME, Evan GI, Elias L (2015). Ibrutinib exerts potent antifibrotic and antitumor activities in mouse models of pancreatic adenocarcinoma. Cancer Res.

[138] Patnaik A, Swanson KD, Csizmadia E, Solanki A, Landon-Brace N, Gehring MP, Helenius K, Olson BM, Pyzer AR, Wang LC (2017). Cabozantinib eradicates advanced Murine prostate cancer by activating antitumor innate immunity. Cancer Discov.

[139] Barkan D, El Touny LH, Michalowski AM, Smith JA, Chu I, Davis AS, Webster JD, Hoover S, Simpson RM, Gauldie J, Green JE (2010). Metastatic growth from dormant cells induced by a Col-I enriched fibrotic environment. Cancer Res.

[140] El Touny LH, Vieira A, Mendoza A, Khanna C, Hoenerhoff MJ, Green JE (2014). Combined SFK/MEK inhibition prevents metastatic outgrowth of dormant tumor cells. J Clin Invest.

[141] Rintoul RC, Sethi T (2002). Extracellular matrix regulation of drug resistance in small-cell lung cancer. Clin Sci (Lond).

[142] Skogseth H, Holt RU, Larsson E, Halgunset J (2006). Tyrosine kinase inhibitors alter adhesivity of prostatic cancer cells to extracellular matrix components. APMIS.

[143] Ribas A, Hersey P, Middleton MR, Gogas H, Flaherty KT, Sondak VK, Kirkwood JM (2012). New challenges in endpoints for drug development in advanced melanoma. Clin Cancer Res.

[144] Voena C, Menotti M, Mastini C, Di Giacomo F, Longo DL, Castella B, Merlo MEB, Ambrogio C, Wang Q, Minero VG (2015). Efficacy of a cancer vaccine against ALK-rearranged lung tumors. Cancer Immunol Res.

[145] Morrissey KM, Yuraszeck TM, Li CC, Zhang Y, Kasichayanula S (2016). Immunotherapy and novel combinations in oncology: current landscape, challenges, and opportunities. Clin Transl Sci.

[146] McDermott DF, Atkins MB, Motzer RJ, Rini BI, Escudier BJ, Fong L, Joseph RW, Pal SK, Sznol M, Hainsworth JD (2017). A phase II study of atezolizumab (atezo) with or without bevacizumab (bev) versus sunitinib (sun) in untreated metastatic renal cell carcinoma (mRCC) patients (pts). J Clin Oncol.

[147] Ribas A, Hodi FS, Lawrence DP, Atkinson V, Starodub A, Carlino MS, Fisher RA, Long GV, Miller WH, Huang Y (2016). Pembrolizumab (pembro) in combination with dabrafenib (D) and trametinib (T) for BRAF-mutant advanced melanoma: phase 1 KEYNOTE-022 study. J Clin Oncol.

[148] Eichelberg C, Heuer R, Chun FK, Hinrichs K, Zacharias M, Huland H, Heinzer H (2008). Sequential use of the tyrosine Kinase inhibitors Sorafenib and Sunitinib in metastatic renal cell carcinoma: a retrospective outcome analysis. Eur Urol.

[149] Farsaci B, Donahue RN, Coplin MA, Grenga I, Lepone LM, Molinolo AA, Hodge JW (2014). Immune consequences of decreasing tumor vasculature with antiangiogenic tyrosine kinase inhibitors in combination with therapeutic vaccines. Cancer Immunol Res.

[150] Reiter K, Polzer H, Krupka C, Maiser A, Vick B, Rothenberg-Thurley M, Metzeler KH, Dorfel D, Salih HR, Jung G, et al. Tyrosine kinase inhibition increases the cell surface localization of FLT3-ITD and enhances FLT3-directed immunotherapy of acute myeloid leukemia. Leukemia. 2017;32:313-22. 10.1038/leu.2017.257 PMC5808080 28895560

[151] Ahn MJ, Sun JM, Lee SH, Ahn JS, Park K (2017). EGFR TKI combination with immunotherapy in non-small cell lung cancer. Expert Opin Drug Saf.

[152] Bendell JC, Kim TW, Goh BC, Wallin J, Oh D-Y, Han S-W, Lee CB, Hellmann MD, Desai J, Lewin JH (2016). Clinical activity and safety of cobimetinib (cobi) and atezolizumab in colorectal cancer (CRC). J Clin Oncol.

[153] Sullivan RJ, Gonzalez R, Lewis KD, Hamid O, Infante JR, Patel MR, Hodi FS, Wallin J, Pitcher B, Cha E (2017). Atezolizumab (A) + cobimetinib (C) + vemurafenib (V) in BRAFV600-mutant metastatic melanoma (mel): Updated safety and clinical activity. J Clin Oncol.

[154] Chowdhury S, McDermott DF, Voss MH, Hawkins RE, Aimone P, Voi M, Isabelle N, Wu Y, Infante JR (2017). A phase I/II study to assess the safety and efficacy of pazopanib (PAZ) and pembrolizumab (PEM) in patients (pts) with advanced renal cell carcinoma (aRCC). J Clin Oncol.

[155] Bhatia S, Curti B, Ernstoff MS, Gordon M, Heath EI, Miller WH, Puzanov I, Quinn DI, Flaig TW, VanVeldhuizen P (2014). Recombinant interleukin-21 plus sorafenib for metastatic renal cell carcinoma: a phase 1/2 study. J Immunother Cancer.

[156] Jackman D, Pao W, Riely GJ, Engelman JA, Kris MG, Jänne PA, Lynch T, Johnson BE, Miller VA (2010). Clinical definition of acquired resistance to epidermal growth factor receptor tyrosine Kinase inhibitors in non–small-cell lung cancer. J Clin Oncol.

[157] Lin Y, Wang X, Jin H (2014). EGFR-TKI resistance in NSCLC patients: mechanisms and strategies. Am J Cancer Res.

[158] Cheng H, Terai M, Kageyama K, Ozaki S, McCue PA, Sato T, Aplin AE (2015). Paracrine effect of NRG1 and HGF drives resistance to MEK inhibitors in metastatic uveal melanoma. Cancer Res.

[159] Li F, Zhu T, Cao B, Wang J, Liang L (2017). Apatinib enhances antitumour activity of EGFR-TKIs in non-small cell lung cancer with EGFR-TKI resistance. Eur J Cancer.

[160] Gainor JF, Shaw AT, Sequist LV, Fu X, Azzoli CG, Piotrowska Z, Huynh TG, Zhao L, Fulton L, Schultz KR (2016). EGFR mutations and ALK rearrangements are associated with low response rates to PD-1 pathway blockade in non-small cell lung cancer: a retrospective analysis. Clin Cancer Res.

[161] Haratani K, Hayashi H, Tanaka T, Kaneda H, Togashi Y, Sakai K, Hayashi K, Tomida S, Chiba Y, Yonesaka K (2017). Tumor immune microenvironment and nivolumab efficacy in EGFR mutation-positive non-small-cell lung cancer based on T790M status after disease progression during EGFR-TKI treatment. Ann Oncol.

[162] Lam W, Jiang Z, Guan F, Huang X, Hu R, Wang J, Bussom S, Liu SH, Zhao H, Yen Y, Cheng YC (2015). PHY906(KD018), an adjuvant based on a 1800-year-old Chinese medicine, enhanced the anti-tumor activity of Sorafenib by changing the tumor microenvironment. Sci Rep.

[163] Tan HY, Wang N, Man K, Tsao SW, Che CM, Feng Y (2015). Autophagy-induced RelB/p52 activation mediates tumour-associated macrophage repolarisation and suppression of hepatocellular carcinoma by natural compound baicalin. Cell Death Dis.

[164] Tan HY, Wang N, Tsao SW, Che CM, Yuen MF, Feng Y (2016). IRE1alpha inhibition by natural compound genipin on tumour associated macrophages reduces growth of hepatocellular carcinoma. Oncotarget.

